# Milk exosomes elicit a potent anti-viral activity against dengue virus

**DOI:** 10.1186/s12951-022-01496-5

**Published:** 2022-07-06

**Authors:** Vengala Rao Yenuganti, Sumbul Afroz, Rafiq Ahmad Khan, Chandrima Bharadwaj, Deepti Kailash Nabariya, Nagaraj Nayak, Madhuri Subbiah, Kumaraswami Chintala, Sharmistha Banerjee, Pallu Reddanna, Nooruddin Khan

**Affiliations:** 1 Department of Animal Biology, School of Life Sciences, University of Hyderabad, Hyderabad, Telangana India; 2 Department of Biotechnology and Bioinformatics, School of Life Sciences, University of Hyderabad, Hyderabad, Telangana India; 3 National Institute of Animal Biotechnology (NIAB), Hyderabad, Telangana India; 4 Department of Bio Chemistry, School of Life Sciences, University of Hyderabad, Hyderabad, Telangana India

**Keywords:** Goat milk exosomes, Dengue virus, Anti-viral, And NDV-K

## Abstract

**Background:**

Exosomes are nano-sized vesicles secreted by various cells into the intra and extracellular space and hence is an integral part of biological fluids including milk. In the last few decades, many research groups have proved the potential of milk exosomes as a sustainable, economical and non-immunogenic drug delivery and therapeutic agent against different pathological conditions. However, its anti-viral properties still remain to be unearthed.

**Methods:**

Here, we have been able to isolate, purify and characterize the milk derived exosomes from Cow (CME) and Goat (GME) and further studied its antiviral properties against Dengue virus (DENV), Newcastle Disease Virus strain Komarov **(**NDV-K) and Human Immunodeficiency Virus (HIV-1) using an in-vitro infection system.

**Results:**

TEM, NTA and DLS analysis validated the appropriate size of the isolated cow and goat milk exosomes (30–150 nm). Real-time PCR and immunoblotting results confirmed the presence of several milk exosomal miRNAs and protein markers. Our findings suggest that GME significantly decreased the infectivity of DENV. In addition, we confirmed that GME significantly reduces DENV replication and reduced the secretion of mature virions. Furthermore, heat inactivation of GME did not show any inhibition on DENV infection, replication, and secretion of mature virions. RNase treatment of GME abrogates the anti-viral properties indicating direct role of exosomes in DENV inhibition. In addition GME inhibited the infectivity of NDV-K, but not HIV-1, suggesting that the GME mediated antiviral activity might be virus specific.

**Conclusion:**

This study demonstrates the anti-viral properties of milk exosomes and opens new avenues for the development of exosome-based therapies to treat viral diseases.

**Graphical Abstract:**

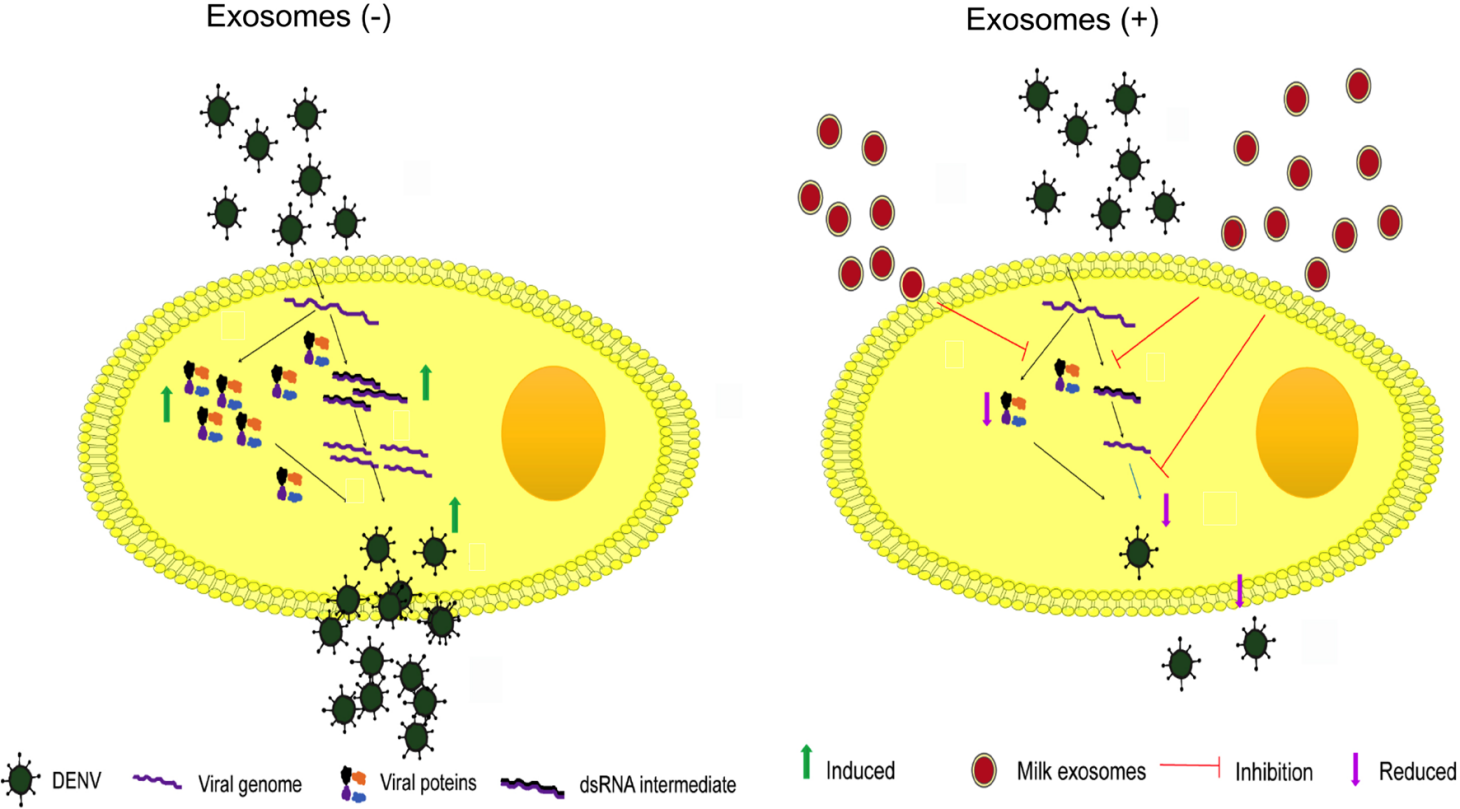

**Supplementary Information:**

The online version contains supplementary material available at 10.1186/s12951-022-01496-5.

## Introduction

Dengue is a mosquito borne viral disease caused by dengue virus (DENV) infecting over 300 million people every year. It is prevalent in more than 100 countries and all the four serotypes of DENV (DENV-1, 2, 3, and 4) are known to be infectious, but the extent of pathogenicity varies. According to World Health Organization (WHO), there is a continuous upsurge in the number of DENV cases to many folds in the last twenty years which is attributed to various factors like rainfall, global warming, urbanization, globalization, and inefficient control of the mosquito vector *Aedes aegypti* [1]. The existing treatment strategy for DENV infection is the symptomatic treatment of patients at different stages of Dengue. Patients with Dengue Fever (DF) are continuously monitored for platelet count, hematocrit and hemorrhagic manifestations. The lines of treatment for patients with Dengue Hemorrhagic Fever (DHF) include rehydration therapies, continuous administration of intravenous fluids and plasma expanders to reduce rapid plasma leakage [2]. The unavailability of a potent DENV vaccine till date has made the eradication of this rapidly expanding disease even more challenging. Attempts made to develop an efficient tetravalent DENV vaccine have failed due to poor immunogenicity, insufficient B-cell memory responses and insufficient production of antibodies that can neutralize all four DENV serotypes with greater efficiency [3]. Dengvaxia, a live attenuated licensed vaccine mounts a limited protective immunity against the DENV [4]. An upsurge in the number of infections, minimal treatment policies and numerous challenges with candidate live or attenuated tetravalent vaccines have made the exploration of alternate strategies the need of the hour. Other RNA viruses which are difficult to cure due to non-availability of vaccines and therapies are HIV-1 and NDV-K. Human Immunodeficiency Virus-1 (HIV-1) is an acquired immunodeficiency syndrome (AIDS) causing virus that belongs to retroviridae family and lentivirus genus. Naturally, host is equipped with restriction factors to block various steps of viral replication and these factors provide first line of defense upon viral entry. However, HIV-1 by counteracting these factors successfully infects and invades the host. Further, the failure in the generation of vaccine and inefficiency of combined antiretroviral therapy (cART) against HIV-1 urged scientific community to re-visit and develop the strategies to fight against this deadly pathogen [5–9]. As an alternative strategy, it was recently shown that exosomes can be used as successful nano vehicles to deliver viral transcription repression components [10]. It will be interesting to see if the exosomes from different non-human sources also possess anti-HIV-1 viral activity.

Newcastle Disease (ND) is a highly contagious disease of birds infecting more than 250 avian species across the world causing substantial economic loss to the poultry industry [11, 12]. ND is rated as one of the most economically important viral diseases as it affects the bird’s health and indirectly leads to food and economic losses. The causative agent of ND is NDV, an enveloped virus with a single stranded, non-segmented negative-sense RNA genome of approximately 15 kb size [13]. Vaccines that are available do not confer desired effectiveness. There are no specifically recommended antiviral drugs for use against NDV. The use of synthetic anti-virals is not economically feasible [14]. Hence, there is a need to look out for effective anti-viral agents from natural sources for use against DENV, HIV-1 and NDV.

Exosomes are membranous nano-structures (30–150 nm) secreted by numerous mammalian cells and therefore found in the various body fluids including blood, cerebrospinal fluid, milk, and urine. Exosomes encapsulate different biomolecules like proteins, lipids, mRNAs, and miRNAs. Exosomes can modulate various cell function either by delivering the functional active molecules or binding the receptors and activating different signaling pathways. They may regulate different stages of cancer; facilitate distant cell communication, direct microbial pathogenesis, and immune modulations [15–20]. The vital role of extracellular vesicles in various viral infections is well documented [21]. Exosomes are present in various body fluids capable of transporting various biomolecules, which make them an attractive tool for developing new diagnostic, drug delivery and therapeutic interventions [22–25]. Emerging findings categorically implicate the therapeutic relevance of exosomes in several biomedical applications, including the control of viral diseases [24–26]. Exosomes can be purified from a wide variety of biological sources, including milk [23, 27–29]. It is a well-established fact that milk-derived exosomes safeguard miRNAs from harsh conditions like low acidic condition, digestive process and RNase treatment [30, 31]. Further, exosomes derived from colostrum milk contain many proteins implicated in immune regulation, growth, repair, and development of infants [32]. Accumulating evidences indicate a potential role of milk-derived exosomes as transporters of miRNAs for eliciting regulatory functions in the recipient cells [33]. Further, Näslund et al. [9] showed that human breast milk exosomes significantly inhibited HIV-1 infectivity in monocyte-derived dendritic cells [9]. However, the effect of milk exosomes on viral infections such as Dengue is not well known. Therefore, in the present study, we investigated the anti-viral properties of milk exosomes against the DENV, NDV-K, and HIV-1 viruses.

## Results

### Biophysical and molecular characterization of cow and goat milk derived exosomes

**Fig. 1:**
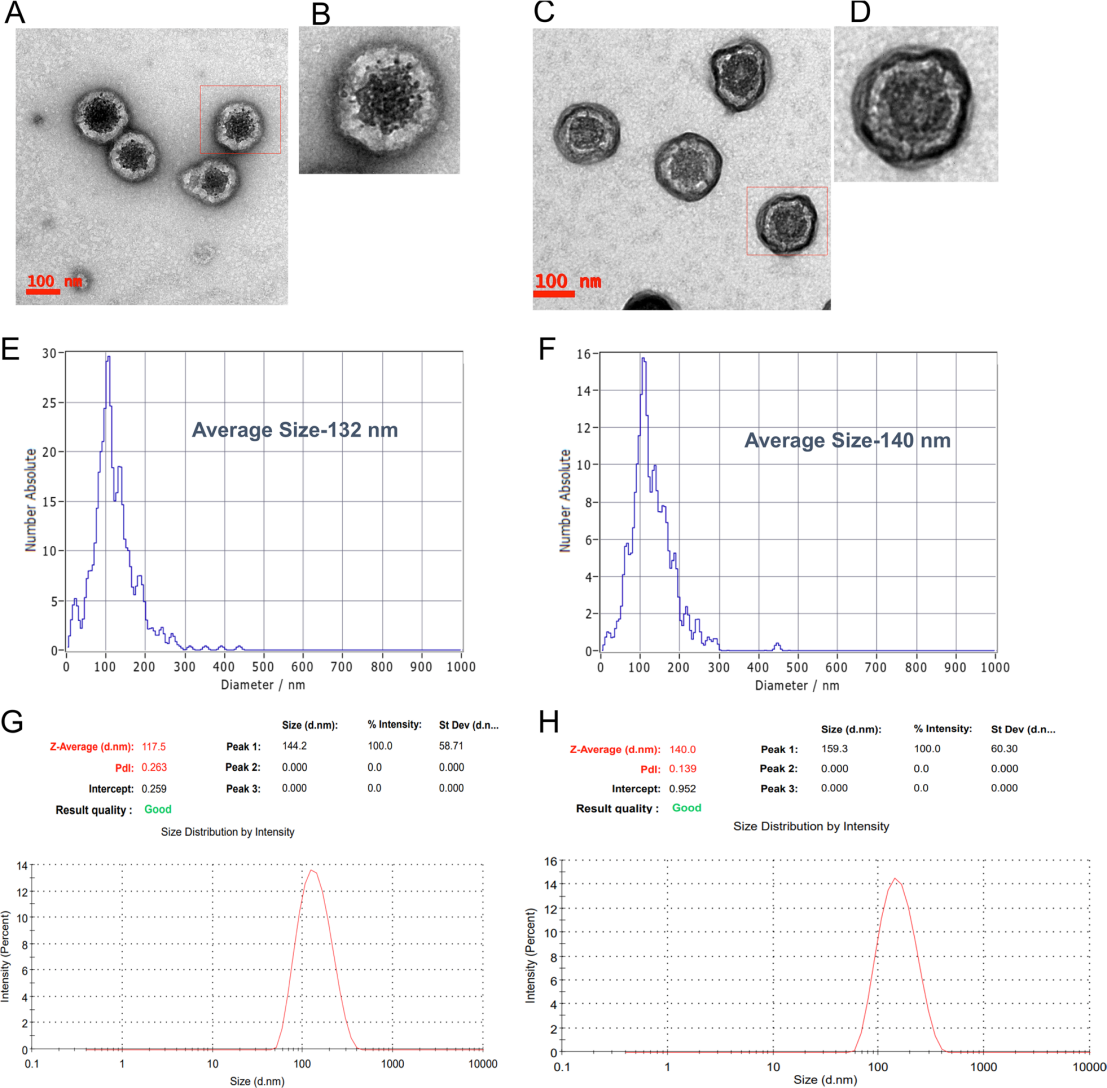
Biophysical characterization of milk exosomes. **A**–**D** Transmission electron microscopy image of GME (**A** and **B**) and CME (**C** and **D**). **E**–**H** Size distribution of GME and CME determined by ZetaView (**E** and **F**) and DLS (**G** and **H**)

**Fig. 2:**
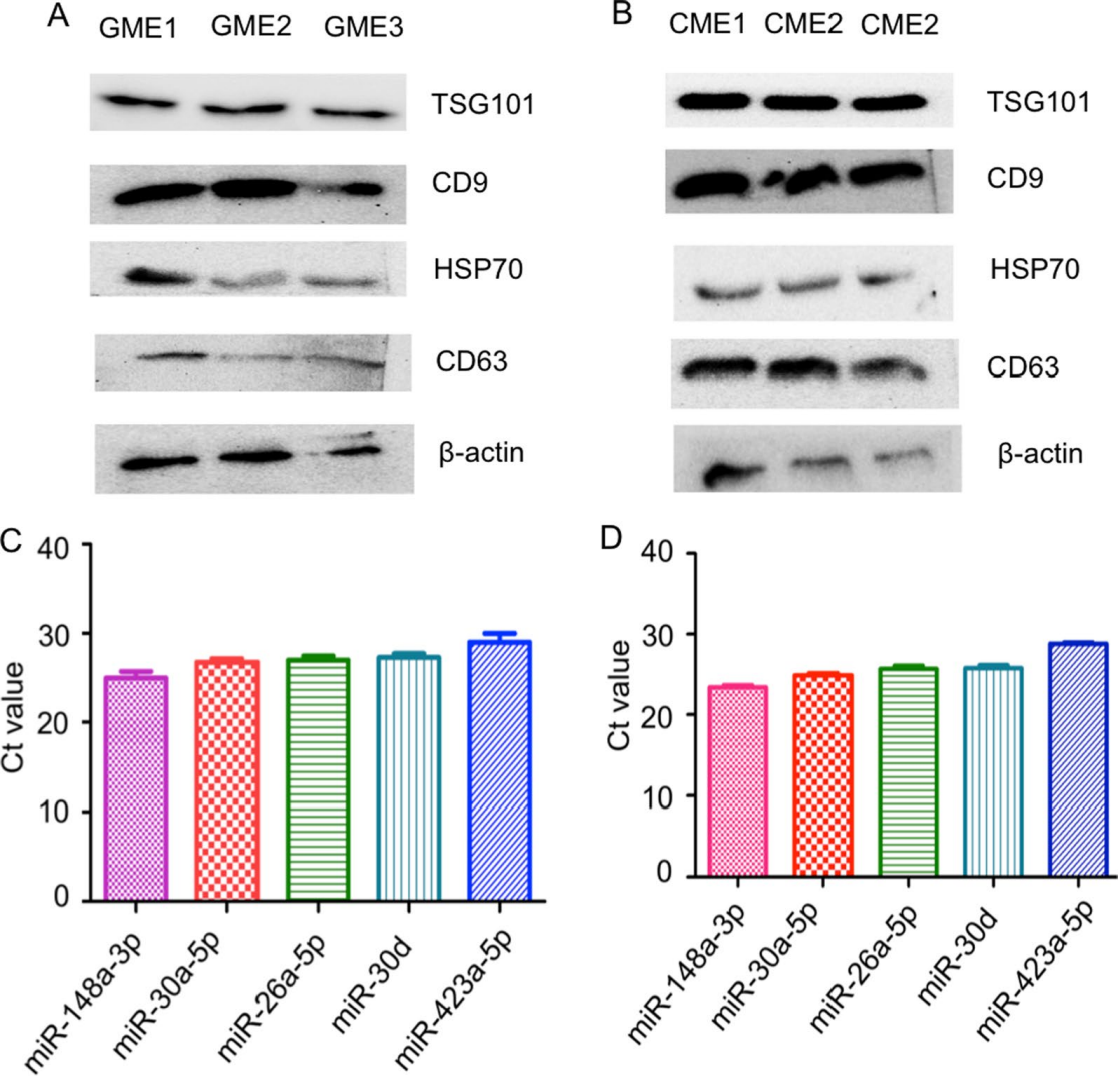
Molecular characterization of GME by immunoblotting and Real-time PCR. **A**, **B** Identification of exosomal markers (TSG101, CD9, HSP70, CD63, and ß-actin) in the GME (**A**) and CME (**B**) by western blotting. **C**, **D** Identification of different milk exosomal miRNAs in the GME (**C**) and CME (**D**) by Real-time PCR. GME1, GME2, and GME3 indicate Goat milk exosomes sample-1, Goat milk exosomes sample-2, and Goat milk exosomes sample-3 respectively. CME1, CME2, and CME3 indicate Cow milk exosomes sample-1, Cow milk exosomes sample-2, and Cow milk exosomes sample-3, respectively

Analysis of cow milk exosomes (CME) and goat milk exosomes (GME) by TEM, DLS and NTA clearly show that the size of the exosomes isolated from the goat and cow milk varied from 30–150 nm (Fig. 1A–H). Immuno-blotting of goat and cow milk exosomes suggested the presence of exosomal markers, TSG101, CD9, Hsp70, CD81 CD63 and β-actin (Fig. 2A, B). In addition to this, real-time PCR results revealed the presence of mir-148a, miR-26a-5p, miR-30a-5p, miR-30d, and miR-423-5p (Fig. 2C, D).

### GME shows a significant anti-viral effect against DENV and NDV-K but not against HIV-1 infection

**Fig. 3:**
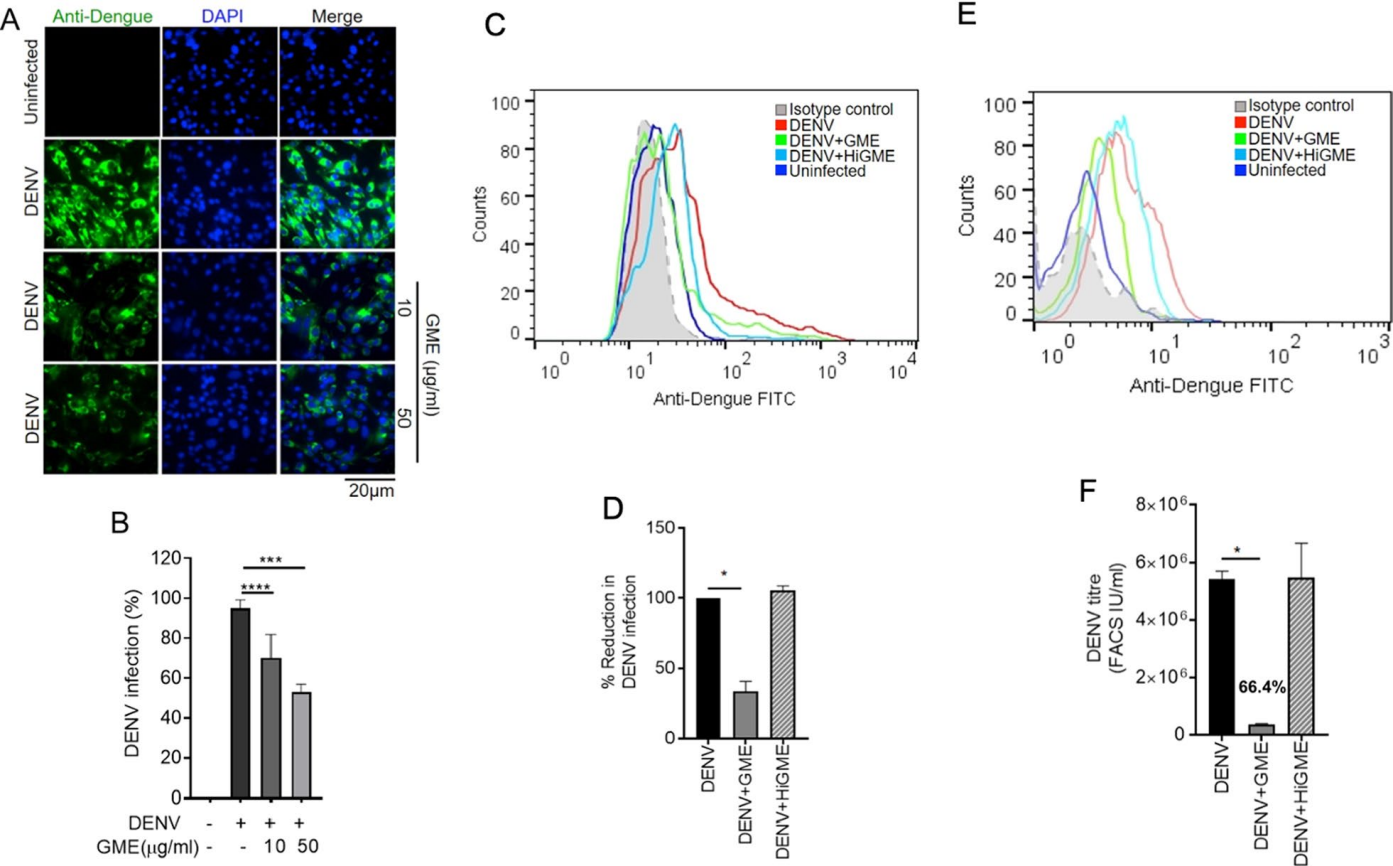
GME show anti-viral effect against DENV. **A** Vero cells were infected with DENV-2 (moi 2) virus for 48 h. Immunofluorescence image presents the number of DENV-2 positive Vero cells treated with two doses of GME. **B** Bar graph showing percentage of DENV-2 positive cells quantified using Image J (NIH) software. Data is represented as mean ± SD from ten dissimilar fields of three independent experiments. **C**–**F** Vero cells were infected with DENV-2 (moi 2) virus and cultured with 50 µg/ml goat exosomes (GME) or 50 µg/ml of heat inactivated goat exosomes (HiGME) for 48 h. Following incubation, the culture supernatant was collected. DENV-infection was analyzed in the cells while the infectious viral yield was analyzed in culture supernatants. **C** Histogram FACS plot showing DENV-2 infection in cells. **D** Bar graph showing the percentage of reduction in DENV-2 infection upon treatment with goat exosomes in DENV-2 infected Vero cells. Data is mean ± SD from three independent experiments. *P < 0.05, ***P < 0.001, ****P < 0.0001 was considered significant. **E** Fresh Vero cells were infected with culture supernatants for 24 h and viral yield/titre was determined through FACS. Histogram FACS plot showing DENV-2 infection. **F** Bar graph displays the viral titre (FACS IU/ml) in the culture supernatants. Data is mean ± SD from three independent experiments. Statistical analysis through Mann Whitney* t*-test (**B**) and two –tailed unpaired Student’s* t* test (**D**,** F**)

After the physical and molecular characterization of the purified exosomes, we demonstrated the anti-viral property of milk exosomes using cultured Vero cells infected with DENV 2 followed by treatment with different concentrations of CME and GME for 24 and 48 h. DENV infection was then determined by analyzing NS3 expression. We observed a dose-dependent inhibition in DENV-2 infection, demonstrated by the reduction in DENV-2 NS3 protein expression after 48 h of GME treatment. In comparison, not much reduction was observed in NS3 expression after 24 h of GME treatment, indicating that GME is effective against DENV at later time-points (Additional file 1: Fig. S1A). Although exosomes derived from cow showed a dose-dependent reduction in DENV-2 NS3 protein levels, they were less efficient than GME in inhibiting DENV-2 NS3 expression (Additional file 1: Fig. S1A). To rule out the possibility of cell death by exosomes, we then analyzed the cytotoxic level of GME on Vero cells through MTT based cell viability assay. The results indicate that GME was not cytotoxic to cells up to 100 µg/ml concentration (Additional file 1: Fig. S1B). To determine the effective treatment of GME on DENV infectivity, we first cultured Vero cells with non-toxic concentrations of GME, 6 h prior to the virus infection, after which the Vero cells were subjected to DENV 2 infection for 48 h (pretreatment). In the case of post-treatment, the cells were initially infected with the DENV 2, after which the GME was added to the cells and cultured for 48 h. The DENV-2 infection was monitored by detecting the levels of DENV NS3 protein through immuno-blotting. Our results clearly showed that pretreatment and post-treatment of GME significantly reduced the expression of the NS3 protein. However, the inhibition was much more significant when GME was added to the medium after DENV infection (Additional file 1: Fig. S1C). All the remaining experiments were conducted by treating the cells with GME post-infection. To further test the anti-viral properties of GME, Vero cells were infected with DENV-2 (moi 2) virus and treated with two doses of GME for 48 h and the DENV infectivity was analyzed in Vero cells through immunofluorescence microscopy. The immunofluorescence microscopy images showed a dose-dependent decrease in the percentage of DENV positive cells upon treatment with GME when compared to only DENV infected cells (Fig. 3A, B). Flow cytometry was also performed to determine the extent of inhibition of infection by GME. We infected Vero cells with DENV-2 virus for 48 h with or without GME and collected the culture supernatant post incubation. We analyzed the viral infectivity in cells and infectious viral yield in the culture supernatant. Our results showed a significant reduction in DENV-2 infection in Vero cells upon GME treatment when compared to DENV-2 infected cells alone (Fig. 3C, D). Further, there was no inhibition in DENV infection after treatment with heat-inactivated GME (HiGME), validating the anti-DENV function of GME (Fig. 3C, D). Heat inactivation of exosomes was carried out by incubating them at 95 °C for 30 min. For estimating the infectious viral yield/titre in the culture upon GME treatment, supernatant of DENV-2 infected cells treated with or without GME was taken. Fresh Vero cells were plated overnight and infected with the collected culture supernatant for 90 min. The medium was then removed, and a fresh medium was added to the cells and incubated for 24 h. Our FACS results revealed a 66.4% reduction in viral yield (represented as infectious units/ml) upon GME treatment. However, no reduction in virus yields upon treatment with HiGME (Fig. 3E, F) was observed.

**Fig. 4:**
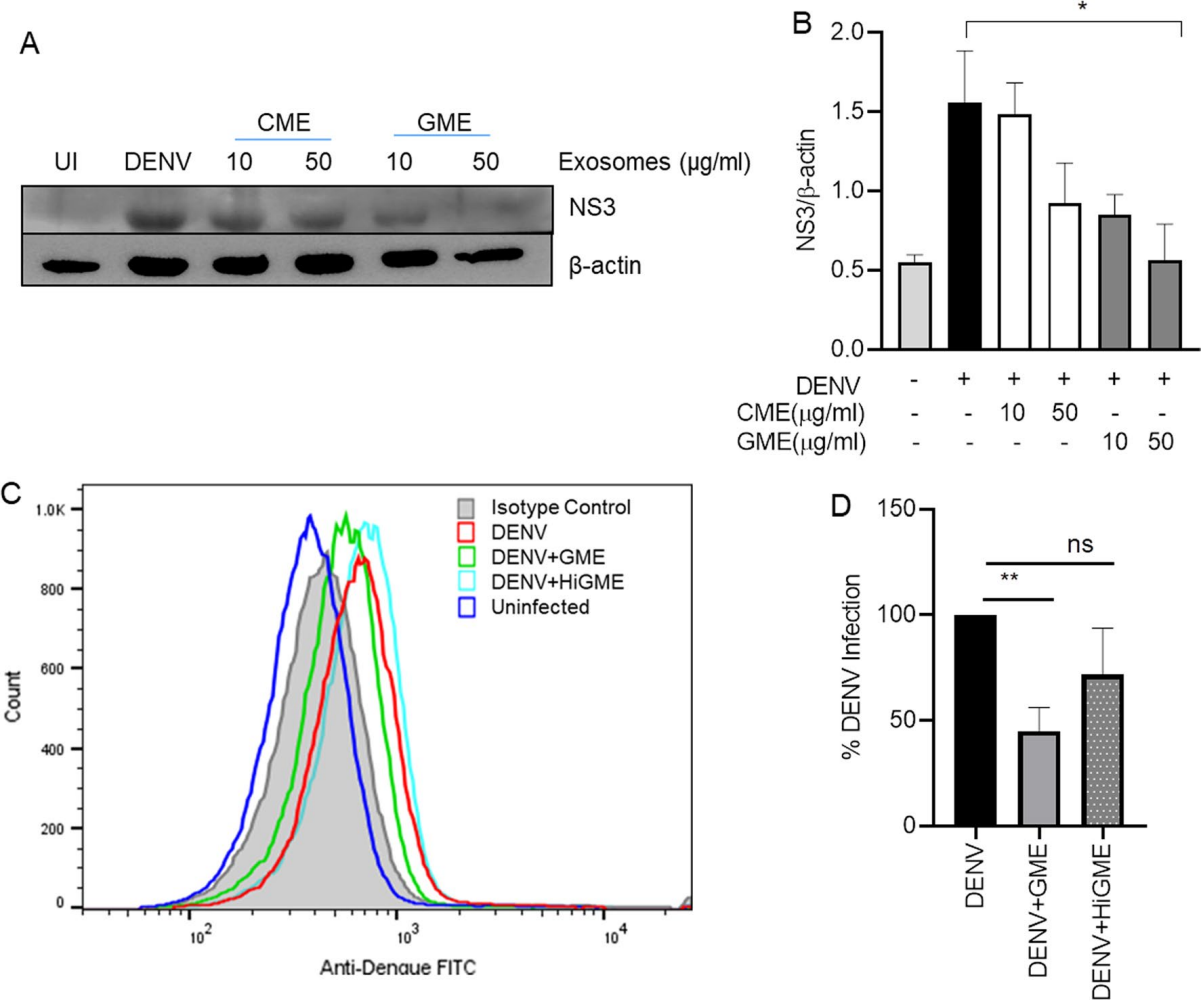
Anti-viral effect of CME and GME on DENV infected megakaryocyte. **A** Immunoblot showing NS3 expression in DENV-2 infected megakaryocytes treated with two different concentrations of GME and CME for 48 hpi. **B** Bar graph showing densitometry analysis of NS3 protein expression from four independent experiments. **C** Histogram FACS plots showing DENV-2 infection in megakaryocytes. **D** Bar graph representing the percentage of normalized DENV-2 infection upon treatment with GME and HiGME in DENV-2 infected megakaryocytes. Data is mean ± SD from three independent experiments

To rule out the possible role of cell death in reduction of viral load due to GME in virus-infected cells, we carried out a cell viability assay, through MTT assay in the DENV infected cells treated with or without GME. Our results showed no cell death (Additional file 1: Fig. S2) at 24 or 48 h post infection, thereby confirming that the reduction in DENV infectivity is due to anti-viral property of GME and not due to cell death. Also, anti-viral activity of GME was tested against different serotypes of DENV. Results demonstrate that GME is effective inhibiting infection against other serotypes of DENV. It showed 56.94%, 30.3% and 32.4% inhibition against DENV-1, DENV-3 and DENV-4, respectively (Additional file 1: Fig. S3A–F). Further to test anti-viral property of GME and CME under physiological conditions, we used DENV human non-differentiated megakaryocyte-erythrocyte progenitor cell line K562. These cells were cultured and differentiated to megakaryocytes by treatment with phorbol 12-myristate 13-acetate (PMA) as reported previously [34]. The differentiated cells were infected with DENV 2 virus (moi 2) followed by treatment with GME or CME for 48 h and infectivity of DENV was analyzed by immunoblotting and flow cytometry. We observed that the GME treatment reduced DENV-2 infection evident from the NS3 immunoblotting and flow analysis, in megakaryocytes which is line with the reduced DENV infectivity observed in the Vero cells (Fig. 4).

After observing the anti-viral properties of GME against DENV, we investigated for their possible ant-viral properties on other RNA viruses, HIV-1 and NDV. To test anti-HIV-1 activity of CME and GME, TZM-bl cells were cultured and treated with CME and GME followed by infection with HIV-1 (pre and post treatment) and infectivity of HIV-1 was analyzed by TZM-bl assay and we observed that both pre and post treatment of CME and GME showed no significant effect on HIV-1 infectivity (Additional file 1: Fig. S4). We also evaluated the antiviral effects GME on NDV-K using Chicken embryonic fibroblast cells (DF-1 cells). The DF-1 cells were cultured and treated with different concentrations of GME. Our results showed about 40% inhibition in Newcastle Disease Virus strain Komarov (NDV-K) infection upon GME treatment at a higher dose (50 μg/ml) (Additional file 1: Fig. S5). These results highlight that GME has significant anti-viral activity against DENV and NDV-K compared to HIV-1. Additionally, exosomes derived from goat milk produced at the 60th day of lactation reduced DENV NS3 expression to a much larger extent when compared to exosomes derived from colostrum and on the 30th day of lactation from goat, indicating that the anti-DENV activity of GME increases with the increase in a lactation period of the goat (Additional file 1: Fig. S6).

### GME inhibits DENV infectivity by interfering with its replication

**Fig. 5:**
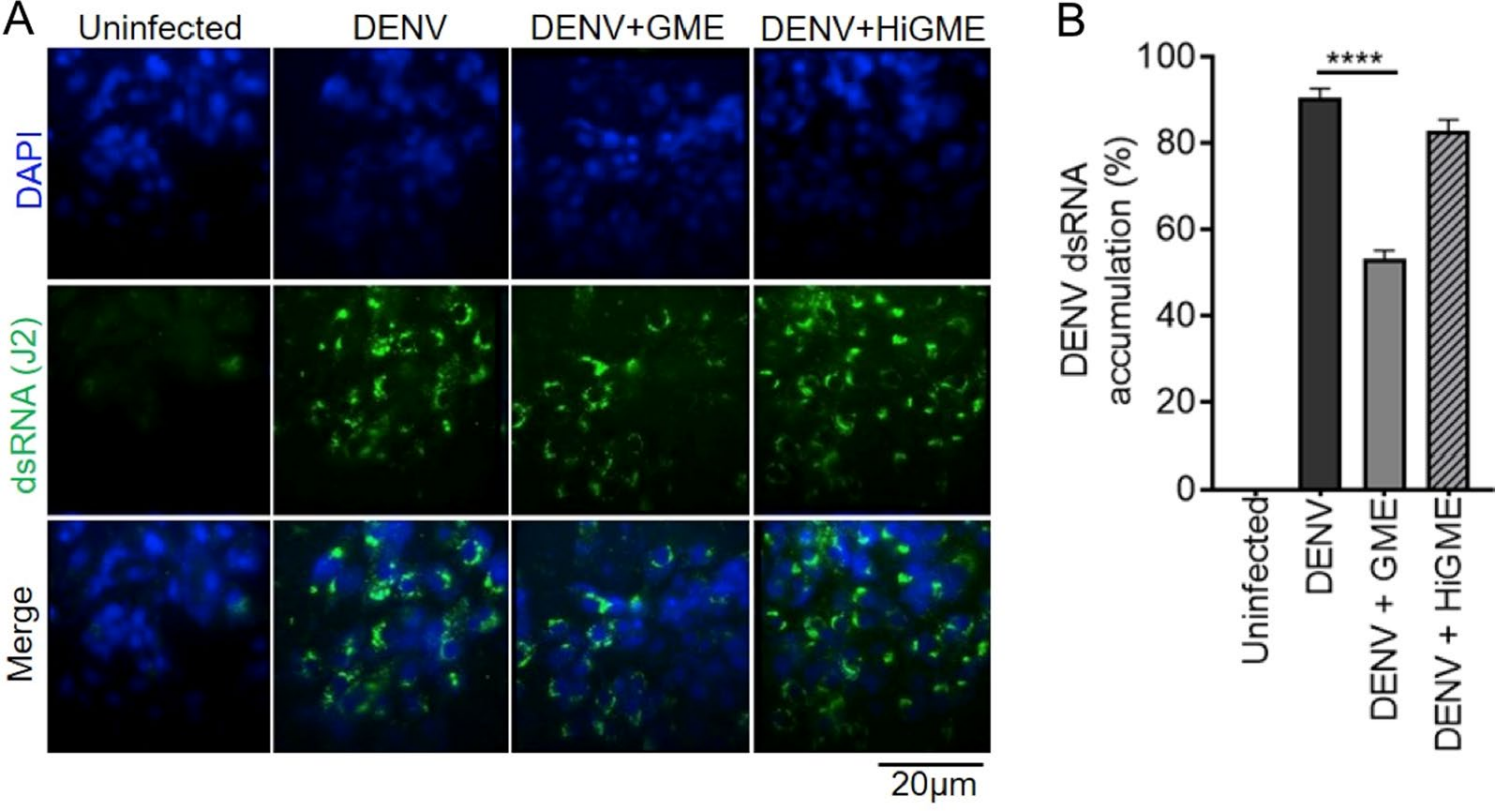
GME shows anti-viral effect on DENV by interfering with its replication. **A** Immunofluorescence image showing the accumulation of dsRNA, stained using anti-dsRNA antibody (green) in DENV-2 infected Vero cells cultured with 50 µg/ml GME or HiGME for 36 h. **B** Bar graph showing dsRNA accumulation in cells quantified through Image J (NIH) software. Data represented as mean ± SD from ten different fields of three independent experiments. ****P < 0.0001 was considered significant. Statistical analysis was done using Mann Whitney t-test

To understand the mechanisms of GME mediated inhibition of DENV infection, we first evaluated its effect on DENV replication. Vero cells were infected with DENV followed by treatment with 50 µg/ml of GME or HiGME for 36 h. Subsequently cells were fixed and stained with J2 antibody for visualizing the accumulation of dsRNA intermediate formed during the DENV replication cycle. Our immunofluorescence results indicate a significant reduction in the accumulation of DENV dsRNA intermediate in the cytoplasm of infected cells treated with GME when compared to only DENV infected cells; however, no inhibition was observed in infected cells treated with HiGME (Fig. 5). These results propose that GME might show an anti-viral effect against DENV by interfering with its replication.

### In-vitro uptake of goat milk exosomes by the cells

**Fig. 6:**
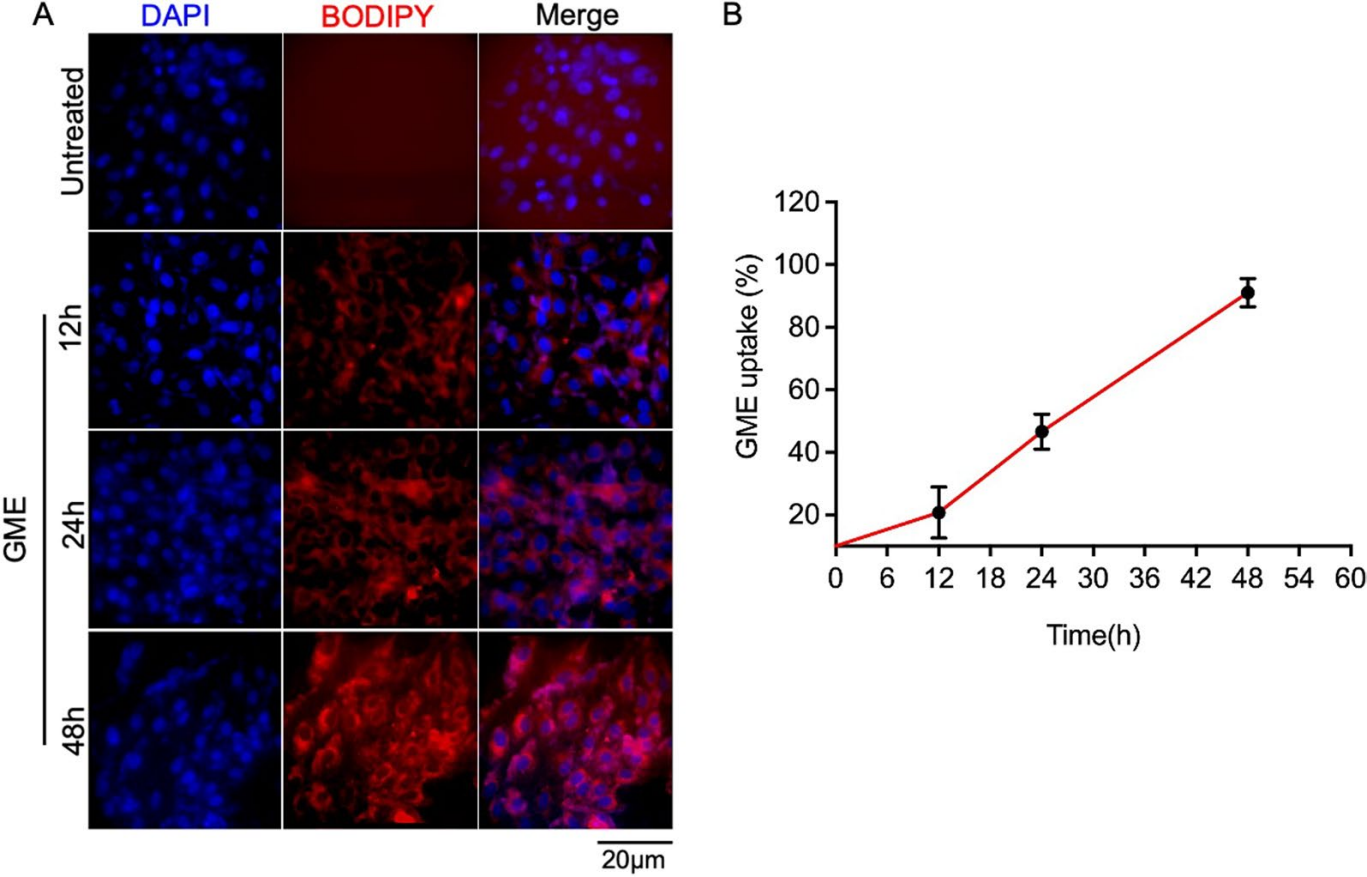
Goat milk derived exosomes uptake by Vero cells. (**A**) Immunofluorescence image showing the uptake of BIODIPY labeled goat exosomes in Vero cells treated with 50 µg/ml of labeled exosomes at indicated time-points. Blue represents the DAPI stained nucleus. (**B**) Graph showing the percentage uptake of labeled exosomes. Quantification of percentage exosome uptake in Vero cells using Image J (NIH) software. Data represented as mean ± SEM from ten different fields of three independent experiments

To examine the kinetics of GME uptake, Vero cells were treated with 50 µg/ml of BODIPY tagged GME for time duration of 12 to 48 h, and GME uptake was visualized through immunofluorescence microscopy. Our results showed a time-dependent uptake of GME by cells, as there was an upsurge in the entry of GME in the cells with the increase in treatment time with more than 90% cells showing red BIODIPY stain at 48 h, suggesting that GME enters into a maximum number of cells within 48 h (Fig. 6).

### Heat treatment of GME alters its morphology and reduces its cellular uptake

**Fig. 7:**
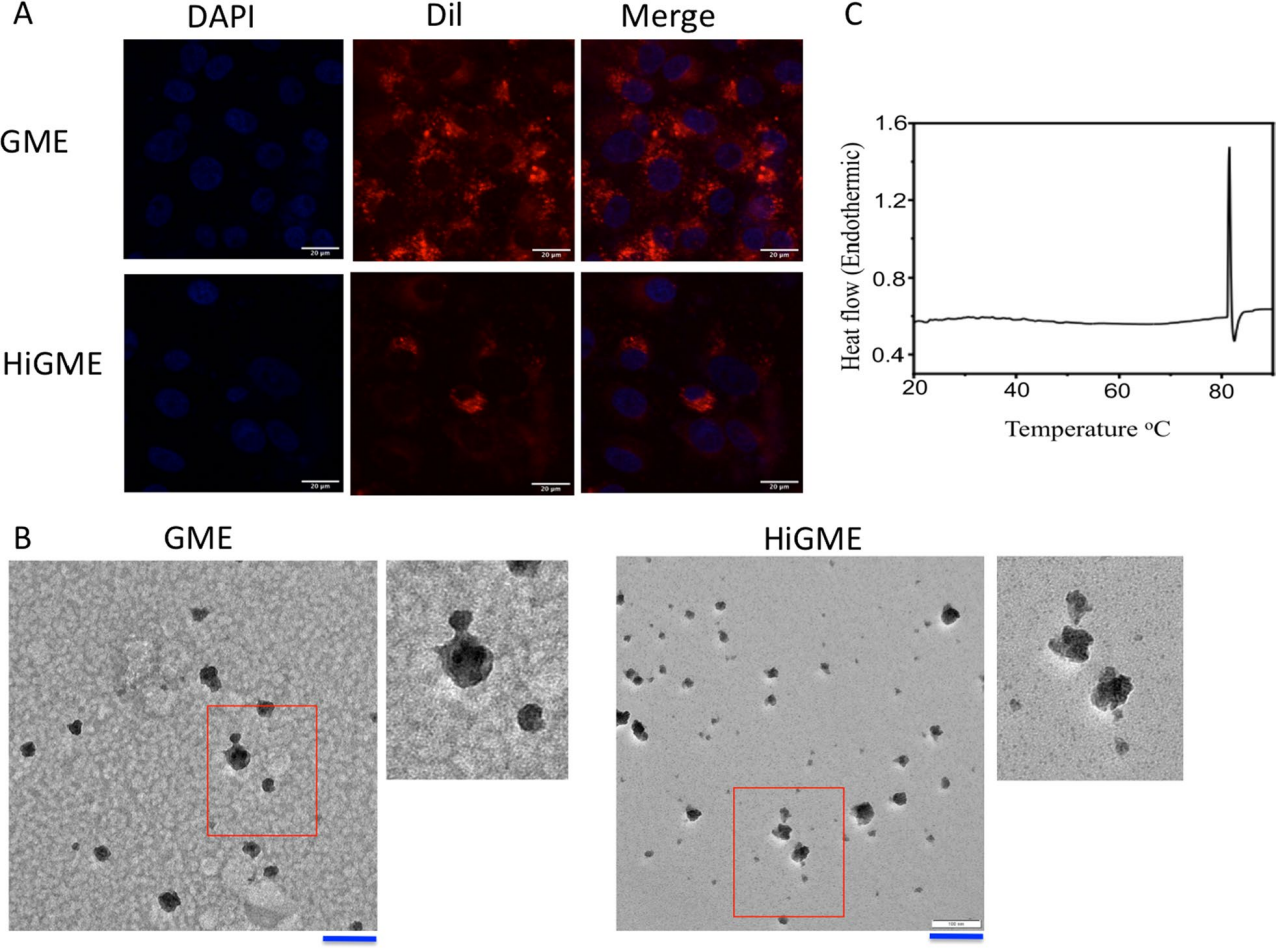
Effect of heat inactivation on GME. **A** Vero cells were incubated with Dil labeled GME and HiGME for 24 h and exosomal uptake was analyzed by fluroescence imaging. Scale Bar- 20 μm. **B** TEM images of normal GME and HiGME. Red box indicated was further magnified next to GME and HiGME images. Scale Bar- 20 μm. **C** Represents stability of exosomes at different temperature measured by Differential Scanning Calorimetry

Results from the TEM demonstrated that morphology of HiGME was altered from round shape to irregular shape as compared with GME. To analyze cellular uptake of exosomes, GME and HiGME were labeled with Dil dye incubated with Vero cells for 24 h. Fluorescence of Dil labeled exosomes was analyzed and results showed that the amount of red fluorescence was significantly high in cells treated with GME than HiGME. Hence, indicating that heat inactivation of GME results in decreased cellular uptake of exosomes (Fig. 7A, B). Thermo stability of GME was analyzed by the Differential scanning calorimetry (DSC), results shows that GME has a sharp endotherm centered at 80.5 ± 0.5 °C. That indicates that GME were only stable up to 81 °C. After this point stability of GME lost stability due to phase transition (Fig. 7C). However, there was no change in the size of GME upon incubation at 37 °C in PBS for 48 h. This indicates GME were stable at physiological temperature (Additional file 1: Fig. S8).

### GME mediated anti-viral activity against DENV is abolished upon proteinase K and RNase treatment

To identify the active component of exosomes responsible for anti-viral activity, exosomes were treated with proteinase K and RNase. DENV-2 infected cells were treated with GME, Proteinase K treated GME and RNase treated GME for 48 h. Flow cytometry analysis demonstrated that GME inhibited the infectivity of DENV whereas proteinase K and RNase treatment reversed the anti-viral effect induced by the GME significantly (Additional file 1: Fig. S7).

## Discussion

Milk has been an essential dietary source globally with enormous benefits in shaping human health through several beneficial components, including proteins, minerals, ions [35]. Emerging reports unfold the abundance of exosomes in the milk, which has been implicated as an essential therapeutic component [23, 36, 37]. Hence, in this study, we purified exosomes from cow and goat milk and examined their anti-viral potential against DENV, HIV-1 and NDV-K. Our results showed that GME exibited anti-viral activity against DENV, which is evident from the decreased expression of multifunctional non-structural protein NS3, which in turn compromises the activity of multiple proteins, including helicase protease, RNA 5′ triphosphatase (RTPase), and adenosine triphosphatase (ATPase) reviewed in Swarbrick et al., 2017 [38]. NS3 plays a vital role in various processes starting from processing of polyprotein, replication and production of mature virions. NS3/NS2B protein complex which has prolytic activity and cleaves viral polyprotein at various regions. Blockade in the NS3-NS2B function results in the disruption of viral replication [39]. The RNA helicase function of NS3 is known to unwind RNA during DENV RNA genome replication that happens through the formation of a double-stranded RNA replicative intermediate [38–40]. Given the multiple role of NS3 in the DENV replication and maturation, it becomes a potential target for design of antiviral drug.

Therefore, down-regulation of NS3 expression by GME seems to impact the replication and production of mature virions. Our findings categorically demonstrate that GME significantly reduces the replication process as well as mature virion formation at 48 h. Our results strengthened these observations and that shows the maximum exosomal uptake by Vero cells at 48 h. NS3 is highly conserved protein in DENV serotypes. Further, our study showed that GME inhibited the infectivity of other serotypes of DENV. GME inhibit the secretion of DENV from infected cells and these results further strengthen the anti-viral role of GME. Also, GME reduce the infectivity of NDV-K without affecting cell survival in vitro. However, it does not display anti-viral activity against HIV-1, thereby suggesting that GME mediated anti-viral potential could be virus specific. These results concur with the report from Conzelmann et al. [26], which potrays that saliva exosomes exhibit anti-viral effects against the ZIKA virus but not on SARS-CoV2 [26]. A critical role of exosomes in barring the DENV infection in cells was further validated through exosomes’ heat inactivation. In these circumstances, exosomes lose their anti-viral properties against DENV. Further analysis reveled that heat treatment of GME leads to morphological changes in shape of GME that results in reduction in the cellular uptake of GME which might be one of the possible reasons for loss of anti-viral activity of GME. To support above observations our results from the DSC revealed that stability of GME was changed after 81 °C. Hence heat treatment at 95 °C for 30 min leads to changes in structure and stability of GME and subsequent loss of anti-viral activity. However, our results from *invitro* study showed GME were stable at physiological temparature (37 °C). Previously similar results were reported from González et al. [41] group regarding stability of GME. This demonstrated that active forms of exosomes were required for exosome-mediated inhibition of DENV infection in Vero cells. Results from the heat treatment, proteinase K treatment and RNAse treatment clearly indicates that RNA play vital role in the inhibition of DENV infection. However, in the case of Proteinase K treatment also GME mediated anti-viral property was abolished, which could be due to the loss of receptors that are involved in the exosomal uptake into cells. It is very well established fact that proteinase K treatment reduces the exosomal uptake by the cells [42]. Very interestingly, RNAse alone treatment completely abolished the anti-viral property of GME despite no proteinase K treatment. It can be speculated that RNAse treated GME may be efficiently uptaken by the cells but does not provide anti-viral activity due to lack of active anti-viral component (RNA). Therefore, this study emphasizes predominantly the role of exosomal RNA as an active anti-viral molecule. Different miRNA profiling studies demonstrate that GME contain different miRNAs that has anti-viral activity. Goat milk exosomes confirmed abundant presence of various miRNAs like miR-148a-3p, miR-30a-5p and miR146b-5p [43, 44]. miRNAs like miR146b-5p, miR-148a and mR-30a-5p display anti-viral properties against HCV [45, 46].

It is well established that milk exosomes are resistant to digestive processes, can cross the intestinal barrier, and reach different parts of the body with no cytotoxicity [28, 29, 47].

Based on the above observations, we infer that upon consumption of milk, exosomes present in milk may cross-intestinal the barrier and reduce the infection by DENV. Our findings demonstrate that goat milk exosomes has a potential to be used as a therapeutic agent for the treatment of different Dengue strains and NDV-K. Having observed that GME targets NS3, which is a highly conserved protein within the flavivirus families like ZIKV and YFV, it further opens up the possibility of using GME as a potential therapeutic agent against flaviviruses. However, these findings merit further investigation to understand the molecular mechanisms through which milk-derived exosomes exhibit anti-viral activity against the Dengue and other viral infections. In conclusion, this study might open an avenue for the development of exosome-based nutraceuticals to treat viral diseases.

## Conclusion

Goat milk exosomes show anti-viral property against DENV by inhibiting the NS3 expression, replication of viral genome and maturation of viral practicals and further opens an avenue for the development of exosome-based nutraceuticals to treat viral diseases.

## Materials and methods

### Milk exosomes isolation

Fresh goat (Osmanbadi) and cow (Gir) milk samples were collected from the nearby goat and cow farms and brought to the laboratory in cold and sterile conditions. Initially, the cow and goat milk was subjected to centrifugation at 3000 *g* for 30 min at 4 °C to remove cell debris, cells and fat. The supernatant was ultracentrifuged at 50,000 *g* for 1 h at 4 °C and transparent plasma obtained was filtered using the 0.22 μm filters. Then, filtered samples were ultracentrifuged at 110,000 *g* for 1 h 10 min at 4 °C (Beckman Coulter Optima MAX-XP, MLA-150 fixed angle rotor). The supernatant was discarded and pellet was suspended in 1X PBS. Then samples were re- ultracentrifuged at 110,000 *g* for 1 h 10 min at 4 °C to concentrate the exosomes. Finally, the pellet was suspended in 1× PBS and filtered through 0.22 μm filters before storing at − 80 °C for further use.

### Protein isolation and western blotting

Exosomal proteins were isolated by adding the RIPA buffer (containing protease inhibitor cocktail) to the exosomal solution obtained by ultracentrifugation in a 1:1 ratio. The solution was vortexed and incubated for 20 min on ice. After incubation, the solution was centrifuged at 12,000 *g* for 15 min at 4 °C. The protein concentration of exosomes was quantified by Bicinchoninic Protein Assay (BCA) according to the manufacturer’s instructions (Pierce™ BCA Protein Assay Kit). A protein standard curve was made with Bovine Serum Albumin (BSA). From the standard curve, the concentration of the proteins in the sample was obtained. After estimation of protein, 50 μg of protein was loaded and run in 12% gel for identification of the exosomal marker protein, HSP70 (Cat# 4872, Cell signaling technology), CD9 (EPR2949, Abcam), CD63 (EPR5702, Abcam), TSG101 (EPR7130(B)), Abcam), CD81 (EPR4244, abcam) and β-actin (A854, Sigma, USA).

### RNA isolation, cDNA preparation and quantification of milk micro RNAs

Total RNA was isolated from the milk exosomes by the Trizol LS as per manufactures instructions. Purity and quantity of RNA was measured by Nano Drop and cDNA was prepared by miScript II RT kit using 200 ng of RNA. Then different milk exosomal markers were quantified by Real-Time PCR (Bio-Rad, CFX96 Touch Real-Time PCR Detection System) using miRNA specific primers.

### Characterization of goat milk exosomes by transmission electron microscope (TEM) and DLS

Purified CME, GME and HiGME were added drop by drop on the copper grids at the concentration of 1 µg/µl. Subsequently, the grids were allowed to dry overnight at room temperature (RT). After overnight incubation, the grids were subjected to negative staining by adding 2.5 µl of 2% uranyl acetate. Then, the morphology of exosomes was captured by using the transmission electron microscope (FEI, Type: 5021/20 Technai G2S-Twin). The size of GME and CME was analyzed by DLS (Zetasizer, Malvern Instruments Limited, UK). Briefly, the exosomes (10 µg) were added in 1 ml of PBS and the hydrodynamic size was analyzed at an equilibration time of 120 s at 25 °C with a 632.8 nm laser beam. The size GME and CME were measured by ZetaView® NTA (Particle Matrix, Germany).

### Cell viability assay

The viability of cells was checked by MTT (3-(4,5-dimethylthiazolyl-2)- 2,5diphenyltetrazolium bromide) assay. Vero cells seeded (1 × 10^4^ cells per well) in a 96 well plate, were treated with different concentrations of GME for 48 h. Post incubation, 25 µl of 5 mg/ml MTT stock in 1× PBS was added and the cells were incubated at 37 °C for 3 h. After incubation, the supernatant was carefully discarded and DMSO was added to solubilize the insoluble formazan crystals inside the cells. Lastly, absorbance was recorded at 570 nm.

### Analysis of the exosomal uptake by Vero cells

To monitor the uptake of milk exosomes, the Vero cells (5 × 10^5^ cells/ml) were plated in a 12 well dish. After reaching the desired confluence, the cells were treated with BODIPY TR labelled GME (50 μg/ml) and incubated at different time intervals i.e. 12, 24 and 48 h at 37 °C in CO_2_ incubator. GME were labelled with a red fluorescent dye called the BODIPY TR ceramide (Molecular Probes, Life Technologies, Invitrogen™) according to Rani et al*.* [31]. At the end of each time point, the medium was discarded and cells were washed with 1X PBS, fixed with 4% paraformaldehyde by incubating for 15 min at RT. The fixed cells were washed and permeabilized by adding 0.2% Triton X-100 (incubated for 20 min). FBS (10%) was added on the coverslips for blocking for 1 h. Finally, the coverslips that contain cells were mounted on glass slides with mounting solution with DAPI stain (Vector laboratories). Finally, the slides were observed under fluorescence microscope to capture images. To see the effect of heat inactivation on cellular uptake of GME by the Vero cells. For this, GME were incubated at 95 °C for 30 min. After this the exosomes were labeled with Dil (Dioctadecyl-3,3,3′,3′-tetramethylindocarbocyanine perchlorate) dye by incubating them for 15 min at RT. Subsequently, Dil labeled normal and heat inactivated exosomes were centrifuged for 110,000 *g* for 1.10 h and pellet was suspended in PBS. The exosomes were filtered by 0.22 μm sterile filters, then cells were treated with Dil labeled GME (50 μg/ml) and HiGME for 24 h. Subsequently, cells were washed with PBS and cells were fixed with 4% para formaldehyde for 15 min. The coverslip was mounted with Vectashield antifade mounting medium (Vector laboratories) containing DAPI. Then cells were observed under microscope.

### Immunofluorescence

The Vero cells were infected with DENV at a moi of 2 for the time points indicated in the legend and cultured with or without GME. Post infection, the cells were fixed with 4% paraformaldehyde followed by a 20 min permeabilization (0.2% Triton-X 100 at RT). Next, 10% FBS was added to the cells for a 1 h at RT for blocking and further incubated with Anti-Dengue1, 2, 3 and 4 (GeneTex) or anti-dsRNA J2 (English and Scientific Consulting Kft.) primary antibodies for 2 h at 37 °C. Then, the cells were incubated with Alexa Fluor-488-green (Invitrogen) secondary antibody at 37 °C for 1 h. Finally, the cells were washed thrice with 1× PBS and then mounted using Vectashield antifade mounting medium containing DAPI. The images were captured by Leica Trinocular immunofluorescence microscope.

### Quantification of viral infection and yield

The quantification of DENV infection in cells and the infectious virus yield/titre in the culture supernatants was carried out through FACS as described previously [48, 49]. Vero cells were seeded overnight at a density of 5 × 10^4^ cells in a 12-well plate and infected with DENV for 90 min, following which viral inoculum was removed, infected cells were washed with 1× PBS and 400 µl of media with or without GME was added and incubated until 48 h. The culture supernatant after the incubation was collected and stored at − 80 °C for further use. The infected cells were then washed with 1× PBS, 4% paraformaldehyde was used for fixation of the cells for 15 min at RT and permeabilized with 1× Permwash buffer (Biolegend) and incubated with Anti-Dengue 1, 2, 3 and 4 (GeneTex) primary antibody diluted in 1× Permwash buffer overnight at 4 °C. After primary antibody incubation, the cells were incubated with anti-mouse Alexa-488-IgG (Invitrogen) for 1 h at 37 °C. After the incubation with the secondary antibody, the cells were washed and resuspended in FACS buffer and acquired on FACS Fortessa. The analysis for identifying DENV positive cells was carried out through Flowjo software. For the estimation of viral yield in culture supernatants, Vero cells were seeded overnight at a density of 5 × 10^4^ cells in a 12-well plate and infected with 350 µl of thawed culture supernatants for 90 min following which culture supernatants were removed, cells were washed and replaced with fresh complete media and incubated for 24 h. Following incubation, the cells were stained for DENV positive cells as mentioned above. The viral titer was calculated using the following formula [48, 49]: FACS infectious units (IU)/ml = [average percent of positive DENV infected cells—average percent of positive mock infected cells) × (total number of cells in well) × (dilution factor)]/(volume of inoculum added to cells).

### Infection of Newcastle Disease Virus strain Komarov (NDV-K) to cells

Chicken embryonic fibroblast cell lines (DF-1) were cultured and maintained in a growth medium (Dulbecco’s modified eagle medium (DMEM) supplemented with 10% fetal bovine serum (FBS) and 1% Penicillin–Streptomycin solution) at 37 °C in a 5% CO_2_ incubator. 3 X 10^5^ DF1 cells were seeded in a six well plate. The cells were infected upon reaching 80% confluency with 0.5 MOI of Newcastle Disease Virus strain Komarov (NDV-K) for 1 h in serum- and antibiotic-free DMEM, at 37 °C in a 5% CO_2_ incubator with occasional shaking. The cells were washed with PBS and taken for further treatment. Different concentrations of exosomes; 50 μg/ml and 10 μg/ml were prepared by diluting an appropriate number of exosomes in DMEM supplemented with 2% FBS in triplicates. Infected cells treated with DMEM and supplemented with 2% FBS without a exosomes served as positive control for infection. Un-infected cells treated with only DMEM without FBS severed as a negative control. Post infection, the cells were washed with phosphate buffered saline (PBS) and incubated with different concentrations of GME for 3 h at 37 °C in a 5% CO_2_ incubator. The cells were later washed with PBS and were incubated for another 48 h in fresh DMEM supplemented with 2% FBS. Post 48 h, the cells were freeze-thawed thrice and the viral load was estimated by plaque assay. The experiment was performed thrice with three different exosome preparations. African green monkey kidney cell line (Vero) was cultured and maintained in growth medium (Dulbecco’s modified eagle medium (DMEM) supplemented with 10% fetal bovine serum (FBS) and 1% Penicillin – Streptomycin solution) at 37 °C in a 5% CO_2_ incubator. 9 × 10^4^ cells were seeded in each well of the 24 well plates. The supernatant collected from each well was serially diluted and the Vero cells were incubated with 100 µl of each dilution in triplicates for 1 h in serum- and antibiotic-free DMEM, at 37 °C in 5% CO_2_ incubator with occasional shaking. The cells were later washed with PBS and an overlay medium containing 0.8% methylcellulose in serum free DMEM supplemented with 1% Penicillin–Streptomycin was added to the cells. The cells were further incubated at 37 °C in a 5% CO_2_ incubator for 6 days. The overlay medium was removed and the cells were fixed in ice-cold 100% methanol for 20 min at 4 °C. The methanol was removed and the wells were stained for plaques with 1% crystal violet. The number of plaques observed in each well was counted and the percentage reduction in the number of plaques in exosomes treated cells was compared to the untreated but infected cells.

### TZM-bl assay

TZM-bl cells were seeded 24 h before infection at 1 × 10^5^ cells per well in 24 well plate and then allowed for binding with HIV-1 NL4.3 (2 ng p24/well) for 3 h at 37 °C with 5% CO_2_. In case of pre-infection treatment, cells were incubated with exosomes (50 μg/ml) for 6 h before infection was given. Cells then were washed once with PBS to remove unbound virus and further incubated for 48 h in DMEM medium containing 10% FBS at 37 ^0^C with 5% CO_2_. In case of post infection treatment, exosomes (50 μg/ml) were added after cells were incubated with virus and treatment continued for 48 h post infection. 48hpi, cells were washed once with PBS and 120 μl of reporter lysis buffer (Cat.# E397A) was added to each well, followed by 2 freeze thaw cycles at -80 ^0^C for cell lysis. Luciferase activity (RLU) in the cell lysates was measured using Luciferase assay reagent (Cat.# E1483) by Luminometer (Turner Biosystems). RLUs were then normalized to the ug protein quantified by BCA method for each sample.

### Heat inactivation, proteinaseK and RNase treatment to GME

Heat inaction or treatment of GME was carried out at 95 °C for 30 min. For Proteinase K treatment, GME (1000 µg) were incubated with Proteinase K (150 µg/ml) for 1 h at 50 °C. RNase tratment was carried out according to Atayde et al. [50]. Briefly, GME (1000 µg) were incubated with 0.1% triton- × 100 for 5 min then Rnase was added and incubated at 37 °C for 20 min. Then all GME samples were ultracentrifuged to remove proteinase K and RNase for 1.10 h at 110,000 *g*. Then supernant was discarded and pellet was resuspneded in PBS. Then GME, HiGME, Proteinase K treated GME and RNAse GME filltered through 0.22 µm before treatment with cells.

### Thermal stability of GME

Thermal stability of GME was carried out in aqueous medium using Nano differential scanning calorimeter (DSC) from TA instruments. GME (200 μg/ml) were prepared in pH 7.4 phosphate buffer saline. GME solution (600 μl) was taken into sample cell and the same volume of 1× PBS was used for reference. Subsequently, GME solution was to heating and cooling scans between 25 °C and 100 °C at the rate 1 °C/min. After completion of the experiment DSC data was analyzed by using Nano DSC analyzer. In addition we analyzed stability of GME at physiological temperature (37 °C) for different time intervals (0, 24 and 48 h) in PBS. Then size of GME was analyzed by the DLS.

## Supplementary Information

**Additional file 1:** **Fig. S1.** GME shows anti-viral effect against DENV at later time-point. (A) Bar graph shows the densitometry analysis of the NS3 blot from three independent experiments. The expression was normalized to loading control β-actin. (B) Vero cells were treated with different concentrations of goat milk exosomes (GME) for 48h. Cytotoxicity of exosomes on Vero cells was assessed by MTT assay. Bar graph represents data from three independent experiments. (C) Vero cells were pre-treated at various concentrations of GME for 6h followed by DENV-2 (moi 2) infection for 48 h or DENV-2 infected Vero cells were treated with different concentrations of GME and incubated for 48h. Immunoblot showing NS3 expression in the above conditions. β-actin was used as the loading control. **Fig. S2.** GME does not induce cell death in virus-infected Vero cells. Bar graph showing percent cell viability in DENV-2 infected Vero cells treated or not treated with GME after 24 and 48hpi. Bar graph represents data from three independent experiments. **Fig. S3.** GME has anti-viral effects against other DENV serotypes. A) Gating strategy for evaluation of DENV infection in cells through FACS. (B-C, E) FACS plots showing DENV-1 (B) (moi 2), DENV-3 (C) (moi 2) and DENV-4 (E) infection in Vero cells treated or not treated with GME. (D) Bar graph showing the percent reduction in DENV-1 and DENV-3 infection upon treatment with GME calculated using FACS data, analyzed through Flowjo software. (F) Bar graph shows percent reduction of DENV-4 infection upon GME treatment. *P<0.05 was measured to be statistically significant. Statistical analysis was done using Mann Whitney t-test. **Fig. S4.** GME and CME do not elicit anti-viral effects against HIV-1. HIV-1 LTR driven luciferase activity show that both pre and post treatment of either GME or CME have no effect on HIV-1 infection. Three independent experiments with three technical replicates were performed. Treatments were normalized to the infected control (No Treatment), which was taken as one. **Fig. S5.** GME elicits anti-viral effects against NDV-K. Effect of GME on NDV-K infection of DF-1 cells. ****P<0.0001 was considered statistically significant. Statistical analysis was done using Mann Whitney t-test. **Fig. S6.** Anti-viral effect of GME enhances with increase in lactation period of goat. (A-D) Molecular characterization of GME derived from different lactation periods colostrum (A), 30 days (B) and 60 days (C) by identifying milk exosomal miRNAs (A, B and C) and exosomal protein markers (CD9, CD63, CD81) (D). (E-J) Physical characterization of GME isolated from various lactation periods by DLS (Particle analyzer, Litesizer 500, Anton Paar) (E-G) and TEM (H-J). (K) Immunoblot showing NS3 expression in DENV-2 infected Vero cells treated with GME derived from goat milk at different lactation period (K). **Fig. S7.** Effect of Proteinase K and RNase treatment on GME anti-viral activity. (A) Histogram FACS plots showing DENV-2 infection in cells. (B) Bar graph represents effect of Proteinase K treated and RNase treated GMEs on DENV-2 infection. Data is mean ± SD from two independent experiments. **Fig. S8.** Stability of GME at physiological temperature in PBS. (A-C) Size distribution of GME suspended in PBS and incubated for 0, 24 and 48h at 37 ^o^C analysed by DLS (Particle analyzer, Litesizer 500, Anton Paar). (D) Bar graph indicates hydrodynamic size of GME after incubation. Data is mean ± SD from two independent experiments. Statistical analysis was done using unpaired t-test.

## Data Availability

All data generated or analyzed during this research are included in this manuscript.

## Abbreviations

ADE: Antibody-dependent enhancement
BCA: Bicinchoninic Protein Assay
CD-9: Cluster of differentiation-9
CD-81: Cluster of differentiation -81
CME: Cow milk exosomes
DAPI: 4′,6-Diamidino-2-phenylindole
DF: Dengue fever
DHF: Dengue hemorrhagic fever
DENV-1: Dengue virus-1
DENV-2: Dengue virus-2
DENV-3: Dengue virus-3
DLS: Dynamic light scattering
DMEM: Dulbecco’s modified eagle medium
dsRNA: Double strand RNA
GAPDH: Glyceraldehyde 3-phosphate dehydrogenase
GME: Goat milk exosomes
FBS: Fetal bovine serum
HCV: Hepatitis c virus
HiGME: Heat inactivated goat milk exosomes
HIV-1: Human immunodeficiency virus-1
HSP-70: Heat stock protein-70
HSC-70: Heat shock cognate protein-70
MTT: 3-(4,5-Dimethylthiazolyl-2)- 2,5diphenyltetrazolium bromide
Moi: Multiplicity of infection
NDV-K: Newcastle disease virus strain komarov
NS3: Nonstructural Protein 3
NTA: Nano tracker analyser
PBS: Phosphate-buffered saline
TEM: Transmission electron microscope
TSG-101: Tumor susceptibility gene 101
VSV-G: Vesicular stomatitis virus G
YFV: Yellow fever virus
ZIKV: Zika virus
