# Supplementary material for: Milk exosomes elicit a potent anti-viral activity against dengue virus
Source: J Nanobiotechnology. 2022 Jul 6;20:317. doi: 10.1186/s12951-022-01496-5 (PMC9258094; doi:10.1186/s12951-022-01496-5)
Supplement: Supplementary file 1 — Additional file 1: Fig. S1. GME shows anti-viral effect against DENV at later time-point. (A) Bar graph shows the densitometry analysis of the NS3 blot from three independent experiments. The expression was normalized to loading control β-actin. (B) Vero cells were treated with different concentrations of goat milk exosomes (GME) for 48h. Cytotoxicity of exosomes on Vero cells was assessed by MTT assay. Bar graph represents data from three independent experiments. (C) Vero cells were pre-treated at various concentrations of GME for 6h followed by DENV-2 (moi 2) infection for 48 h or DENV-2 infected Vero cells were treated with different concentrations of GME and incubated for 48h. Immunoblot showing NS3 expression in the above conditions. β-actin was used as the loading control. Fig. S2. GME does not induce cell death in virus-infected Vero cells. Bar graph showing percent cell viability in DENV-2 infected Vero cells treated or not treated with GME after 24 and 48hpi. Bar graph represents data from three independent experiments. Fig. S3. GME has anti-viral effects against other DENV serotypes. A) Gating strategy for evaluation of DENV infection in cells through FACS. (B-C, E) FACS plots showing DENV-1 (B) (moi 2), DENV-3 (C) (moi 2) and DENV-4 (E) infection in Vero cells treated or not treated with GME. (D) Bar graph showing the percent reduction in DENV-1 and DENV-3 infection upon treatment with GME calculated using FACS data, analyzed through Flowjo software. (F) Bar graph shows percent reduction of DENV-4 infection upon GME treatment. *P<0.05 was measured to be statistically significant. Statistical analysis was done using Mann Whitney t-test. Fig. S4. GME and CME do not elicit anti-viral effects against HIV-1. HIV-1 LTR driven luciferase activity show that both pre and post treatment of either GME or CME have no effect on HIV-1 infection. Three independent experiments with three technical replicates were performed. Treatments were normalized to the infected cont [file 12951_2022_1496_MOESM1_ESM.docx]

**Title: Milk exosomes elicit a potent anti-viral activity against Dengue virus**

Vengala Rao Yenuganti^1*†^, Sumbul Afroz^1,2†^, Rafiq Ahmad Khan^2^, Chandrima Bharadwaj^2^, Deepti Kailash Nabariya^1^, Nagaraj Nayak^3^, Madhuri Subbiah.^3^, Kumaraswami Chintala^4^, Sharmistha Banerjee^4^, Pallu Reddanna^1^, Nooruddin Khan^1,2*^

^1^Department of Animal Biology, School of Life Sciences, University of Hyderabad, Hyderabad, Telangana, India.

^2^Department of Biotechnology and Bioinformatics, School of Life Sciences, University of Hyderabad, Hyderabad, Telangana, India.

^3^National Institute of Animal Biotechnology (NIAB), Hyderabad, Telangana, India.

^4^Department of Bio Chemistry, School of Life Sciences, University of Hyderabad, Hyderabad, Telangana, India.

†  Both contributed equally for the work

* Share equal corresponding authorship

Corresponding authors emails: [vengal.ndri@gmail.com](mailto:vengal.ndri@gmail.com) and [noor@uohyd.ac.in](mailto:noor@uohyd.ac.in)

**Supplementary Data:**

**Fig. S1**

**
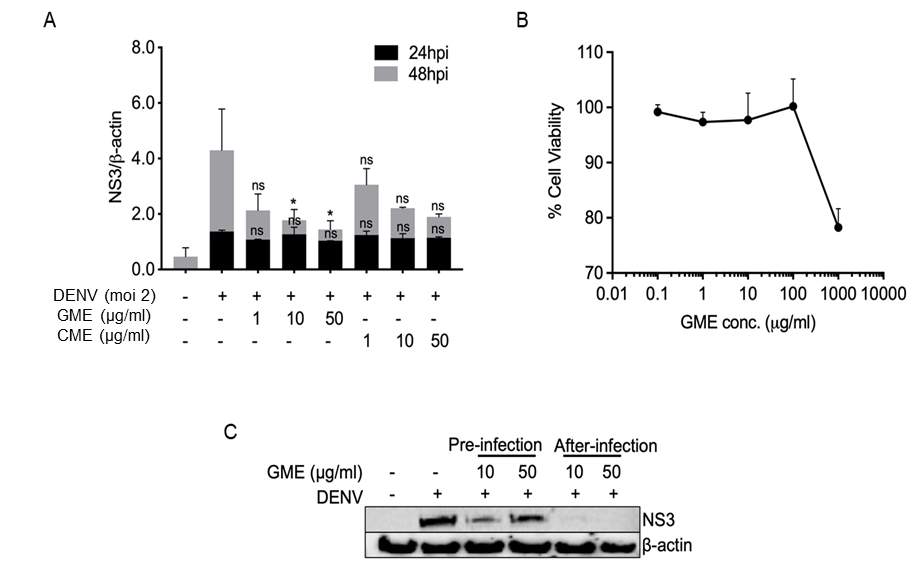
**

**Fig. S1.** **GME shows anti-viral effect against DENV at later time-point.** (A) Bar graph shows the densitometry analysis of the NS3 blot from three independent experiments. The expression was normalized to loading control β-actin. (B) Vero cells were treated with different concentrations of goat milk exosomes (GME) for 48h. Cytotoxicity of exosomes on Vero cells was assessed by MTT assay. Bar graph represents data from three independent experiments. (C) Vero cells were pre-treated at various concentrations of GME for 6h followed by DENV-2 (moi 2) infection for 48 h or DENV-2 infected Vero cells were treated with different concentrations of GME and incubated for 48h. Immunoblot showing NS3 expression in the above conditions. β-actin was used as the loading control.

**Fig. S2**


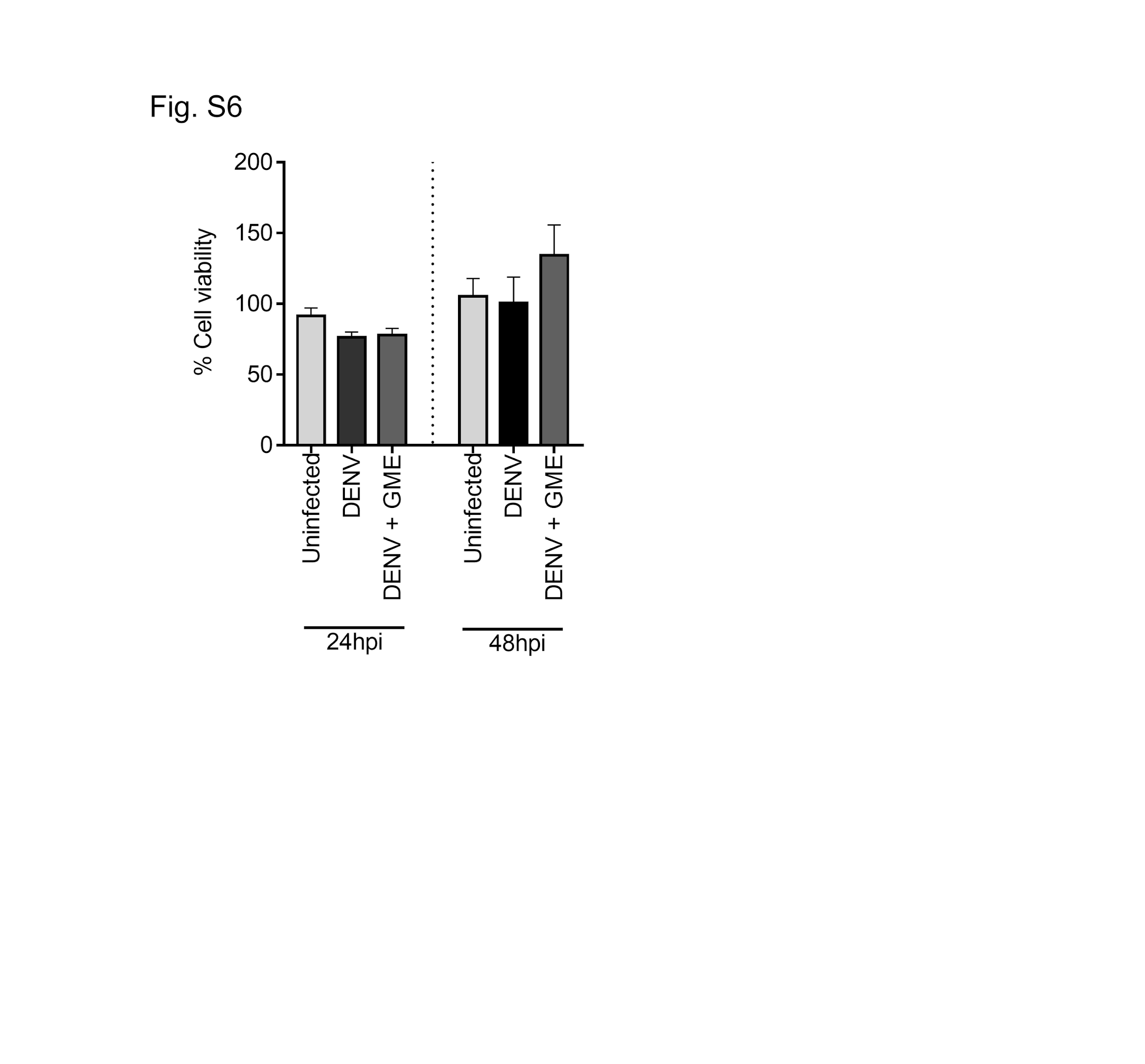


**Fig. S2. GME does not induce cell death in virus-infected Vero cells.** Bar graph showing percent cell viability in DENV-2 infected Vero cells treated or not treated with GME after 24 and 48hpi. Bar graph represents data from three independent experiments.

**Fig. S3**

**
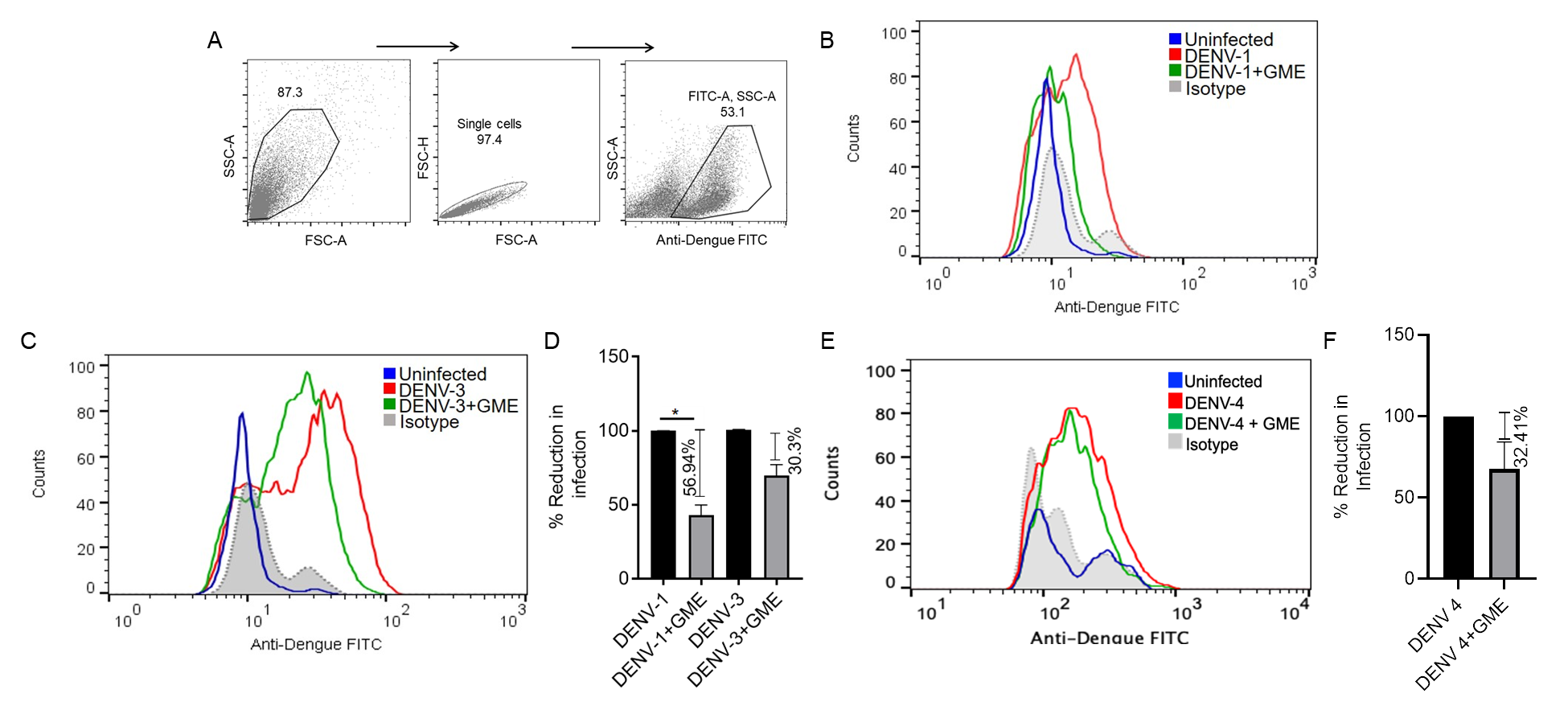
**

**Fig. S3.** **GME has anti-viral effects against other DENV serotypes.** A) Gating strategy for evaluation of DENV infection in cells through FACS. (B-C, E) FACS plots showing DENV-1 (B) (moi 2), DENV-3 (C) (moi 2) and DENV-4 (E) infection in Vero cells treated or not treated with GME. (D) Bar graph showing the percent reduction in DENV-1 and DENV-3 infection upon treatment with GME calculated using FACS data, analyzed through Flowjo software. (F) Bar graph shows percent reduction of DENV-4 infection upon GME treatment. *P<0.05 was measured to be statistically significant. Statistical analysis was done using Mann Whitney t-test.

**Fig. S4**

**
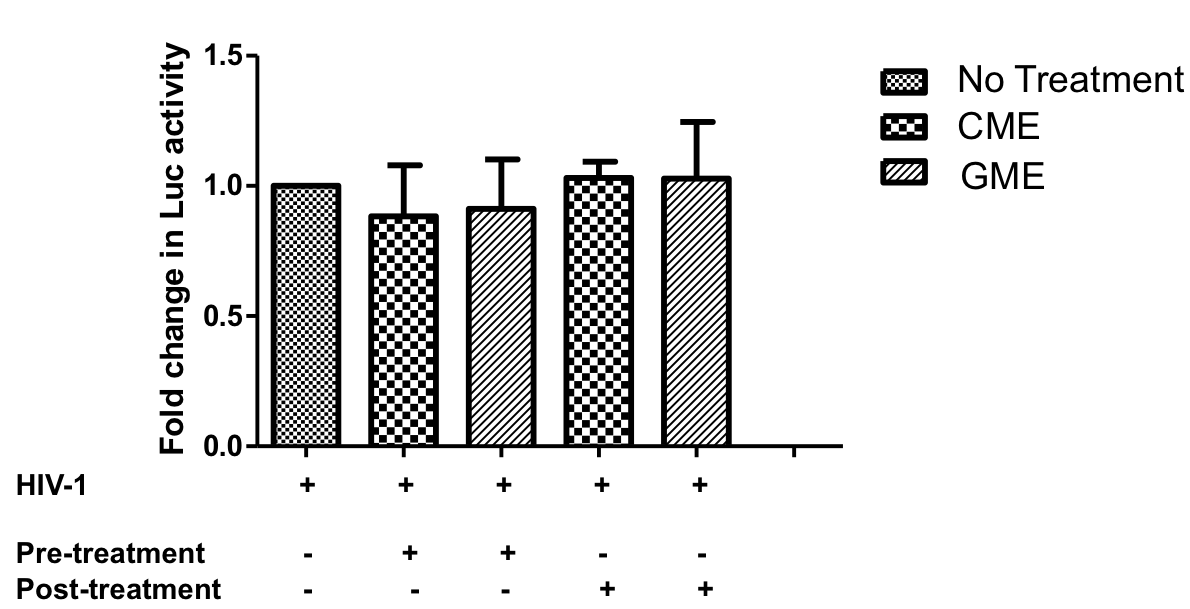
**

**Fig. S4.** **GME and CME do not elicit anti-viral effects against HIV-1.** HIV-1 LTR driven luciferase activity show that both pre and post treatment of either GME or CME have no effect on HIV-1 infection. Three independent experiments with three technical replicates were performed. Treatments were normalized to the infected control (No Treatment), which was taken as one.

**Fig. S5**


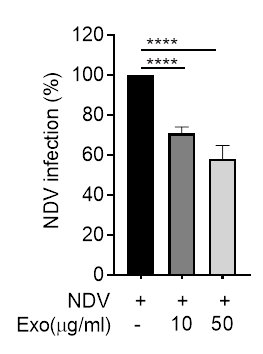


**Fig. S5.** **GME elicits anti-viral effects against NDV-K.** Effect of GME on NDV-K infection of DF-1 cells. ****P<0.0001 was considered statistically significant. Statistical analysis was done using Mann Whitney t-test.

**Fig. S6**


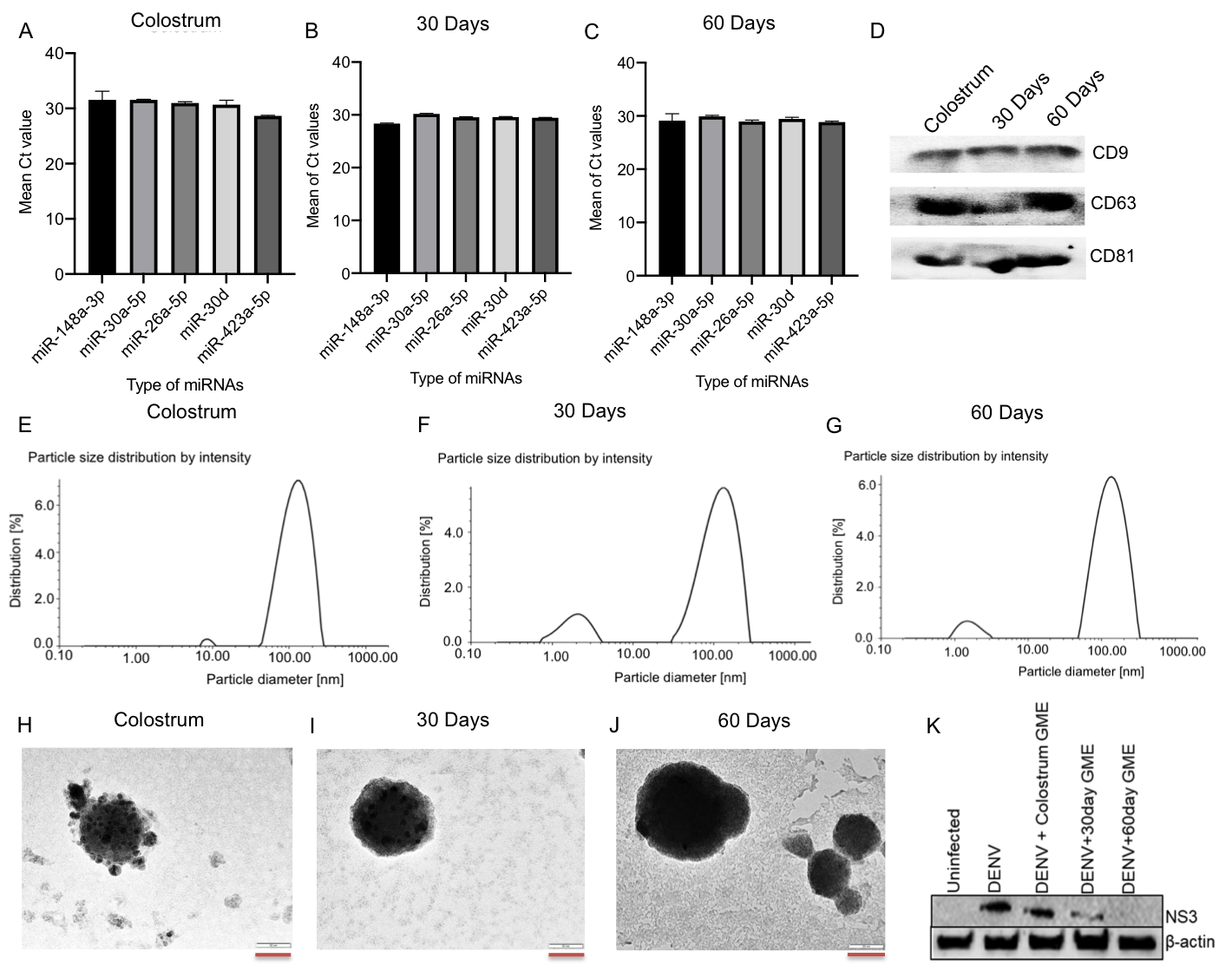


**Fig. S6. Anti-viral effect of GME enhances with increase in lactation period of goat.** (A-D) Molecular characterization of GME derived from different lactation periods colostrum (A), 30 days (B) and 60 days (C) by identifying milk exosomal miRNAs (A, B and C) and exosomal protein markers (CD9, CD63, CD81) (D). (E-J) Physical characterization of GME isolated from various lactation periods by DLS (Particle analyzer, Litesizer 500, Anton Paar) (E-G) and TEM (H-J**)**. **(**K**)** Immunoblot showing NS3 expression in DENV-2 infected Vero cells treated with GME derived from goat milk at different lactation period.

**Fig. S7**


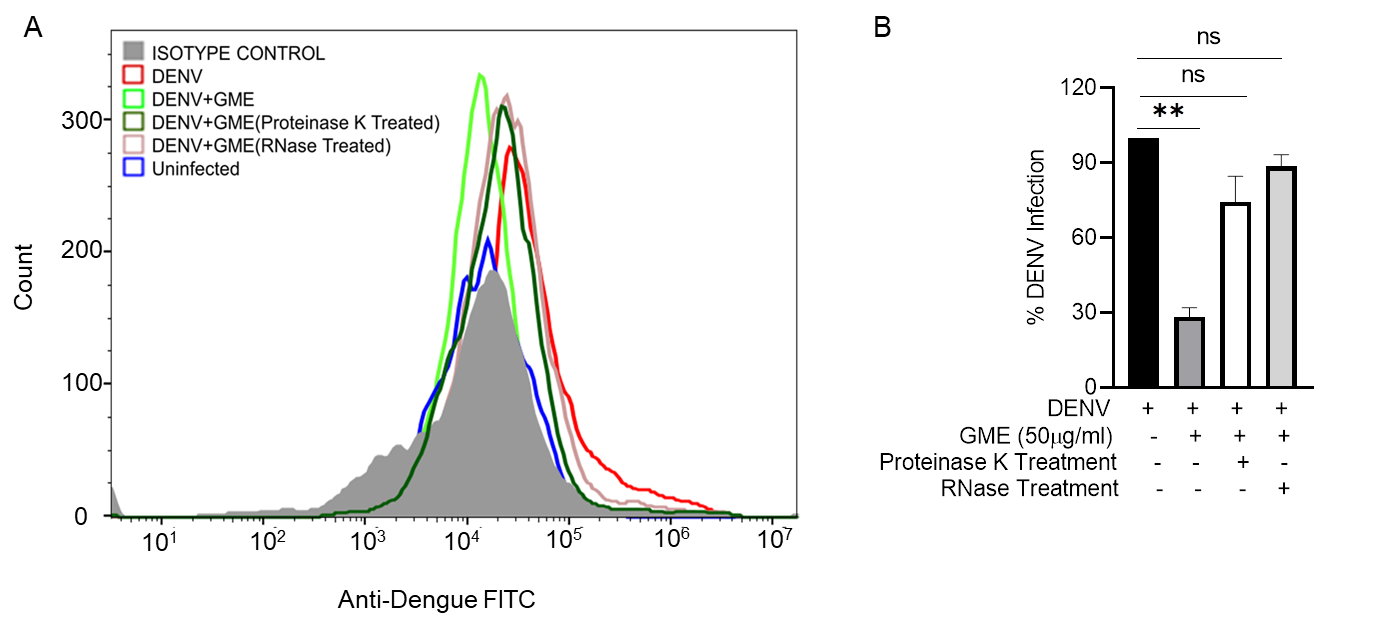


**Fig. S7.** **Effect of Proteinase K and RNase treatment on GME anti-viral activity.** (A) Histogram FACS plots showing DENV-2 infection in cells. (B) Bar graph represents effect of Proteinase K treated and RNase treated GMEs on DENV-2 infection. Data is mean ± SD from two independent experiments.

**Fig. S8**


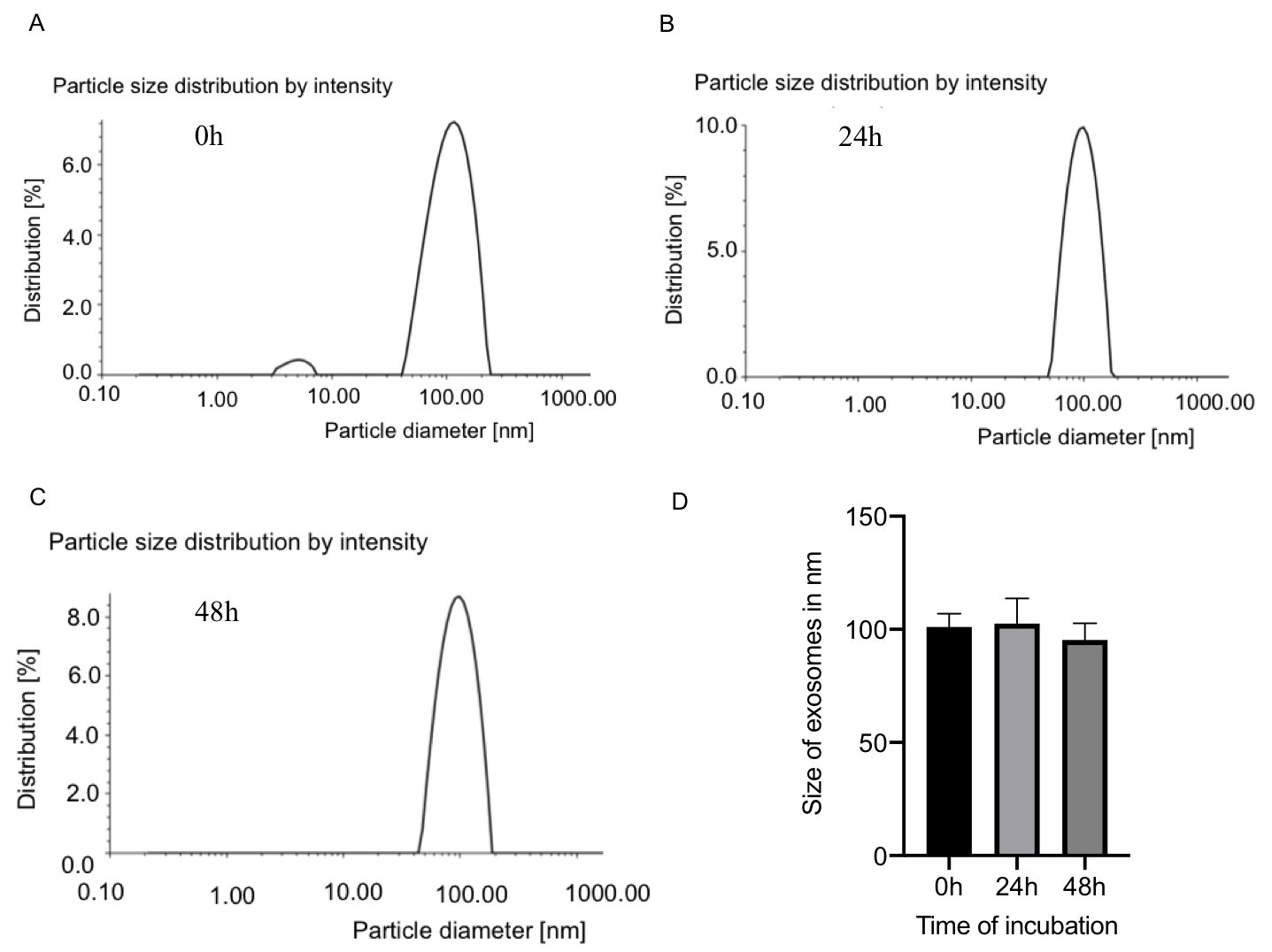


**Fig. S8. Stability of GME at physiological temperature in PBS.** (A-C) Size distribution of GME suspended in PBS and incubated for 0, 24 and 48h at 37 ^o^C analysed by DLS (Particle analyzer, Litesizer 500, Anton Paar). (D) Bar graph indicates hydrodynamic size of GME after incubation. Data is mean ± SD from two independent experiments. Statistical analysis was done using unpaired t-test.
